# A pilot study on comparison of subjective titration versus remotely controlled mandibular positioning during polysomnography and drug-induced sleep endoscopy, to determine the effective protrusive position for mandibular advancement device therapy

**DOI:** 10.1007/s11325-022-02569-3

**Published:** 2022-01-22

**Authors:** Elahe Kazemeini, Sara Op de Beeck, Anneclaire Vroegop, Dorine Van Loo, Marc Willemen, Johan Verbraecken, Marc J. Braem, Olivier M. Vanderveken, Marijke Dieltjens

**Affiliations:** 1 Faculty of Medicine and Health Sciences, Translational Neurosciences, University of Antwerp, Universiteitsplein 1 – D.T.493, 2610, Wilrijk, Antwerp, Belgium; 2 Department of Otorhinolaryngology, Head and Neck Surgery, Antwerp University Hospital, Edegem, Antwerp, Belgium; 3 Multidisciplinary Sleep Disorders Centre, Antwerp University Hospital, Edegem, Belgium; 4 Department of Pulmonary Medicine, Antwerp University Hospital, Edegem, Belgium; 5 LEMP, Faculty of Medicine and Health Sciences, University of Antwerp, Wilrijk, Antwerp, Belgium; 6 Department of Special Dentistry Care, Antwerp University Hospital, Edegem, Antwerp, Belgium

**Keywords:** Oral appliance therapy, Titration, Mandibular protrusion, Remotely controlled mandibular positioner, Sleep-disordered breathing

## Abstract

**Study objectives:**

The aim of this pilot study was to evaluate the clinical effectiveness of subjective titration versus objectively guided titration during polysomnography (PSG) and drug-induced sleep endoscopy (DISE) in mandibular advancement device (MAD) therapy for patients with obstructive sleep apnea (OSA).

**Methods:**

In this pilot cross-over study, patients underwent three titration procedures in randomized order: (1) subjective titration, (2) PSG-guided titration using a remotely controlled mandibular positioner (RCMP) and (3) DISE-assisted titration using RCMP. After each titration procedure, patients used the MAD for 1 month at the targeted protrusion obtained according to the preceding titration procedure. For each procedure, a follow-up PSG was performed after 1 month of MAD use in order to evaluate the efficacy of the MAD.

**Results:**

Ten patients were included in the study. Overall, no significant differences in targeted optimal protrusion compared to maximal comfortable protrusion among the three titration methods were observed. There was no significant difference in reduction in AHI. In this study, PSG titration correctly classified 50% of patients as ‘responder’. A higher predictive accuracy was found for DISE titration with a sensitivity of 83.3% and a specificity of 100%.

**Conclusions:**

This pilot randomized cross-over trial showed no differences in optimal mandibular positioning and corresponding efficacy of MAD between subjective titration, DISE titration or PSG titration.

## Introduction

Obstructive sleep apnea (OSA) is an increasingly common disorder with related socioeconomic healthcare issues. Therefore, effective management of this chronic disorder is imperative [1]. A non-invasive treatment option for OSA is the use of a mandibular advancement device (MAD) during sleep, in order to reduce upper airway collapse by protruding the mandible. MAD should be custom-made and titratable [2, 3]. During a titration procedure, the mandible is gradually positioned more anteriorly.

The amount of protrusion is a key factor in optimizing MAD efficacy. Some studies suggest a dose-dependent effect of mandibular protrusion on the corresponding decrease in apnea–hypopnea index (AHI) [4–6]. However, a recent study showed a non-linear dose-dependent effect, with a plateau stage after reaching approximately 70% of maximal protrusion [7], suggesting that more protrusion does not always yield to a decrease in AHI [4]. Furthermore, to ensure optimal therapy adherence, a compromise between an effective protrusive position and patient tolerance needs to be found. Therefore, it is important to be able to target the protrusive position individually in terms of tolerability versus efficacy [8].

Thus far, no proven standard is available on how to determine such optimal MAD protrusion. Most MAD outcome studies rely on subjective titration (titration_Subj_), wherein the degree of mandibular advancement is progressively increased over several weeks (5 to 40 weeks) until an improvement or a resolution of symptoms occur, or until the patient cannot tolerate any further advancement [2, 9, 10]. Subjective titration does not allow upfront determination of the optimal-protrusion or prediction of treatment outcome. Furthermore, subjective improvement in symptoms is not an accurate indicator for efficient MAD titration and may result in a suboptimal treatment outcome [11].

The mandible may also be progressively protruded during sleep until abolishment of respiratory events, under PSG guidance. Overnight titration has evolved from manual adjustment of a temporary appliance by awakening the patient [6, 11, 12], to titration without awakening the patient by using a hydraulic [13] or motorized advancement system [8, 14, 15], referred to as a remotely controlled mandibular positioner (RCMP). The drawbacks of such a PSG-guided titration (titration_PSG_) are the labor intensiveness, time-consuming nature and need of a sleep technician to work overnight, all limiting the routine applicability of this procedure.

Recently, it was shown that application of an RCMP during drug-induced sleep endoscopy (DISE) is feasible in determining the mandibular protrusion [16]. The DISE procedure is only 45 min in comparison to a full night PSG titration or the period of several weeks for titration_Subj_.

Currently, there are no data available comparing the different titration procedures. The aim of this pilot study was to evaluate the clinical value of titration_Subj_ versus the objectively guided titration during DISE or PSG.

## Material and methods

This clinical trial was registered on Clinicaltrials.gov (NCT03716648) and approved by the local ethical committee at University of Antwerp and Antwerp University Hospital in September 2018 with protocol number 18/33/364 and Belgian Registration number: B300201837436 (protocol version July 2018 dd 08/08/2018). Written informed consent was obtained from all patients prior to the start of the study.

Patients were prospectively recruited by the OSA multidisciplinary team. All patients were diagnosed with OSA (AHI ≥ 15/h) and judged suitable for MAD therapy [17].

The protocol of this prospective, randomized, cross-over study was previously published in detail [18]. Three titration procedures were performed in each patient, in randomized order: (1) Subjective titration (titration_Subj_), (2) PSG-guided titration using RCMP (titration_PSG_) and (3) DISE-assisted titration using RCMP (titration_DISE_). The RCMP device (MATRx, Zephyr Sleep Technologies Inc., Calgary, Canada) uses dental impression trays that fit over the teeth to allow for progressive titration of the mandible during natural sleep (PSG-guided) or induced sleep (DISE-assisted) without arousing the patient. It is controlled by software which activates a stepping motor attached anteriorly to the dental trays [8, 14, 19–21].

All patients were treated with a custom-made, titratable MAD (SomnoDent® Flex™, SomnoMed Ltd, Australia) [2, 22]. After each titration procedure, the patient used the MAD for 1 month in the targeted protrusion obtained according to the preceding titration procedure. Objective adherence was monitored through an embedded microsensor thermometer (TheraMon, IFT Handels- und Entwicklungsgesellschaft GmbH, Handelsagentur Gschladt, Hargelsberg, Austria), assuming that the MAD was worn when a temperature > 35 °C was recorded [23, 24].

A follow-up PSG was performed after 1 month of MAD use to evaluate the efficacy of the MAD in that specific optimal protrusion. A washout interval of 1 week between the consecutive titrations was integrated in the protocol. The same MAD was used for all three titration methods after readjustment to the corresponding position of each titration method.

### Protrusive characteristics

The protrusive characteristics of each patient were assessed by a dental sleep professional. The patient started in habitual occlusion and was then asked to protrude the mandible maximally, known as maximal protrusion (MP). Thereafter, the patient slowly retruded the mandible and then slowly protruded again, until a position was reached that is on the edge of becoming uncomfortable, known as the maximal comfortable protrusion (MCP). The particular setup of the RCMP allows for a limited amount of protrusion if one starts from, e.g. habitual occlusion or centric occlusion. To bypass this limitation, the edge-to-edge position was used as the ‘zero-point’ during the titrationPSG and titration_DISE_. Additionally, the RCMP does not allow any changes in the vertical open distance: the impression trays that are fitted over the dental arcs determine the vertical opening imposed during the determination of the targeted protrusion. So, vertical opening was kept constant for all three methods during this study protocol.

### Arm 1: Subjective titration (titration_Subj_)

The MAD was fitted in the MCP, followed by a 1-month period during which patients were allowed to additionally titrate their MAD. Each patient was individually instructed and trained to perform this titration, until a significant improvement or resolution of subjective symptoms, or until the patient could not tolerate any further advancement. The subjective nature of this titration protocol implies that upfront outcome prediction is not possible as compared to arms 2 and 3 of the study.

### Arm 2: Titration of the mandibular protrusion during a titration_PSG_

At the start of the titration procedure, the dental trays were placed intra-orally over the tooth arcs at the incisor edge-to-edge position of the patient. The titration_PSG_ was performed during a full night attended type 1 PSG using the RCMP device, protruding the mandible in increments of 0.5 and 1.0 mm, without disturbing sleep, and in response to observed apneas and/or hypopneas. If an EEG arousal occurred, no further advancement was attempted until stable sleep resumed. This stepwise mandibular protrusion was continued until either no respiratory events were registered or until the maximal protrusive limit of the patient was reached.

Prediction of treatment outcome was based on the occurrence of respiratory events during the titration night: ‘Predicted success’ was defined as an observation of less than five respiratory events per hour of sleep. If respiratory events remained present while reaching the maximal protrusion, the titration was labeled as ‘predicted failure’. An ‘inconclusive’ event occurred if the titration was prematurely terminated without reaching an effective target protrusion and/or without reaching the maximal protrusion.

If the titration_PSG_ was scored as ‘predicted success’, the MAD was set at the found position, further referred to as the ‘PSG predicted target protrusion’. On the other hand, if the titration_PSG_ was scored as ‘inconclusive’ or as ‘predicted failure’, the MAD was set at 75% of the MP. Patients were instructed not to change this mandibular protrusion over the 1-month period between the titration_PSG_ and the follow-up sleep study. The protrusive position of MAD was controlled at the end of this 1-month period and before the follow-up PSG.

### Arm 3: Titration of the mandibular protrusion during a titration_DISE_

DISE was performed in a semi-dark, silent operating theatre with the patient in the supine position. For the induction of sleep, a single IV bolus of midazolam (1.5 mg) was administered. Subsequently, propofol (2.0–3.0 µg/ml) was administered via a target-controlled infusion pump [25].

The dental trays were placed intra-orally at the edge-to-edge incisor position of the patient prior to the start of the DISE procedure. Thereafter, the flexible fiberoptic nasopharyngoscope was inserted. Titration was then started under direct visualization of the upper airway. The patient’s mandible was protruded in increments of 1 mm in response to the remaining visualized upper airway collapse at various levels of the upper airway, until a stable open airway was maintained. At that point, the titration procedure was fine-tuned in smaller steps of 0.5 mm. Titration was continued until either no upper airway collapses were observed or until the maximal protrusive limit of the patient was reached.

**Fig. 1:**
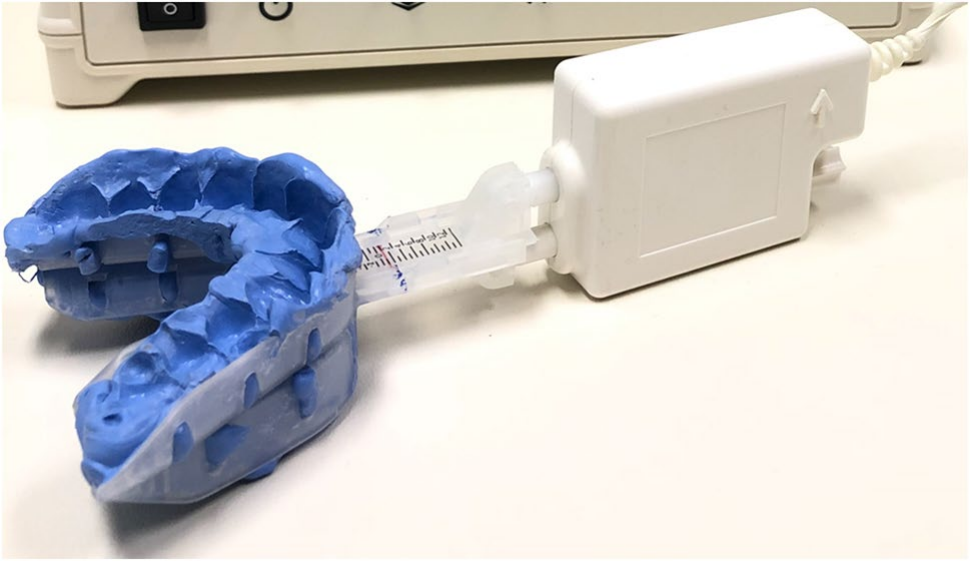
Commercially available remotely controlled mandibular positioner (RCMP). A ruler is incorporated in the RCMP device for visual evaluation of the

After every adjustive movement, the RCMP protrusion and the protrusion of software were doublechecked on the RCMP ruler (Fig. 1), ensuring correct positioning of the mandible during the assessment.

‘Predicted success’ was defined as observation of a stable, open upper airway. The MAD was set at the final position, further referred to as the ‘DISE predicted target protrusion’. If upper airway collapse remained present while reaching the maximal protrusion, the titration_DISE_ was labeled as ‘predicted failure’. An ‘inconclusive’ event occurred if the titration_DISE_ was prematurely terminated without reaching an effective target protrusion and/or without reaching the maximal protrusion. In analogy with the titration_PSG_, the MAD was set at 75% of the MP if the DISE-assisted titrated was scored as ‘inconclusive’ or as ‘predicted failure’.

### Outcomes

The primary outcome was the protrusive positions obtained at the end of the 1-month titration_Subj_ and following RCMP titration during PSG or DISE. The ‘optimal protrusion’ was then calculated as the amount of protrusion or retrusion relative to the MCP, with positive values showing a more forward mandibular position while negative values representing a more retruded position as compared to MCP.

The secondary outcomes included the efficacy of MAD therapy in terms of AHI reduction compared to baseline, as well as the number of responders defined as patients with a reduction in AHI of 50% or more compared to baseline, and/or a follow-up AHI of 10 events/hour or less. Patients were considered lost to follow-up for a specific titration method if the follow-up PSG with the MAD in the titrated protrusion was not performed. Additionally, efficacy of MAD on subjective symptoms like snoring, excessive daytime sleepiness and fatigue was evaluated. A 10-point visual analogue scale (VAS) was used to assess the severity of snoring, with the VAS ranging from 0, representing no snoring, to 10, causing the bed partner to leave the room or sleep separately [26]. The extent of daytime sleepiness was assessed using the Epworth Sleepiness Scale (ESS) [27]. Fatigue severity was assessed using the CIS20R, a 20-item questionnaire that takes approximately 5 to 10 min to complete [28].

A tertiary outcome measure was the objective adherence to MAD therapy for each study arm. Patients were considered ‘compliant’ if the MAD was used ≥ 4 h/night, while ‘regular users’ were defined as patients who used the MAD ≥ 4 h/night on ≥ 70% of all nights.

### Statistical analysis

Statistical analysis was conducted using R software (R Foundation for Statistical Computing, Vienna, Austria). Python (Python software foundation, Centrum voor Wiskunde en Informatica, Amsterdam, Netherlands) was used for creation of the figures. Data were presented as median [Q1; Q3]. A non-parametric paired Friedman test was performed to compare the outcome measures between the different treatment arms. Positive predictive value (PPV) and negative predictive value (NPV) of titration_PSG_ and titration_DISE_ were calculated.

## Results

A total of ten patients were included in this pilot study. Table 1 shows the baseline characteristics. The study population is predominantly male, middle-aged, overweight, with a diagnosis of moderate to severe OSA. Overall, patients experienced mild to moderate hypersomnolence based on ESS score, heavy snoring, and signs of fatigue impeding work-related capacity.

**Table 1:** Baseline characteristics (*n* = 10). *BMI* body mass index, *AHI* apnea–hypopnea index, *OAHI* obstructive AHI, *ODI* oxygen desaturation index, *REM* rapid eye movement, *ESS* Epworth Sleepiness Scale, *VAS* visual analogue scale, *CIS20R* checklist individual strength

Parameter	Median	Q1; Q3
% Male	90%	-
BMI (kg/m^2^)	30.2	27.2; 31.2
Age (years)	48.0	41.5; 55.6
AHI (events/h)	21.3	17.5; 26.8
OAHI (events/h)	18.5	17.4; 24.9
Supine AHI (events/h)	32.8	22.7; 53.6
REM AHI (events/h)	34.1	29.0; 46.7
ODI (events/h)	13.5	12.28; 20.25
Sleep efficiency %	85.0	83.4; 86.1
VAS snoring (0–10)	8	7; 9
ESS (0–24)	13	10; 16
CIS20R (20–140)	87	41; 101

**Fig. 2:**
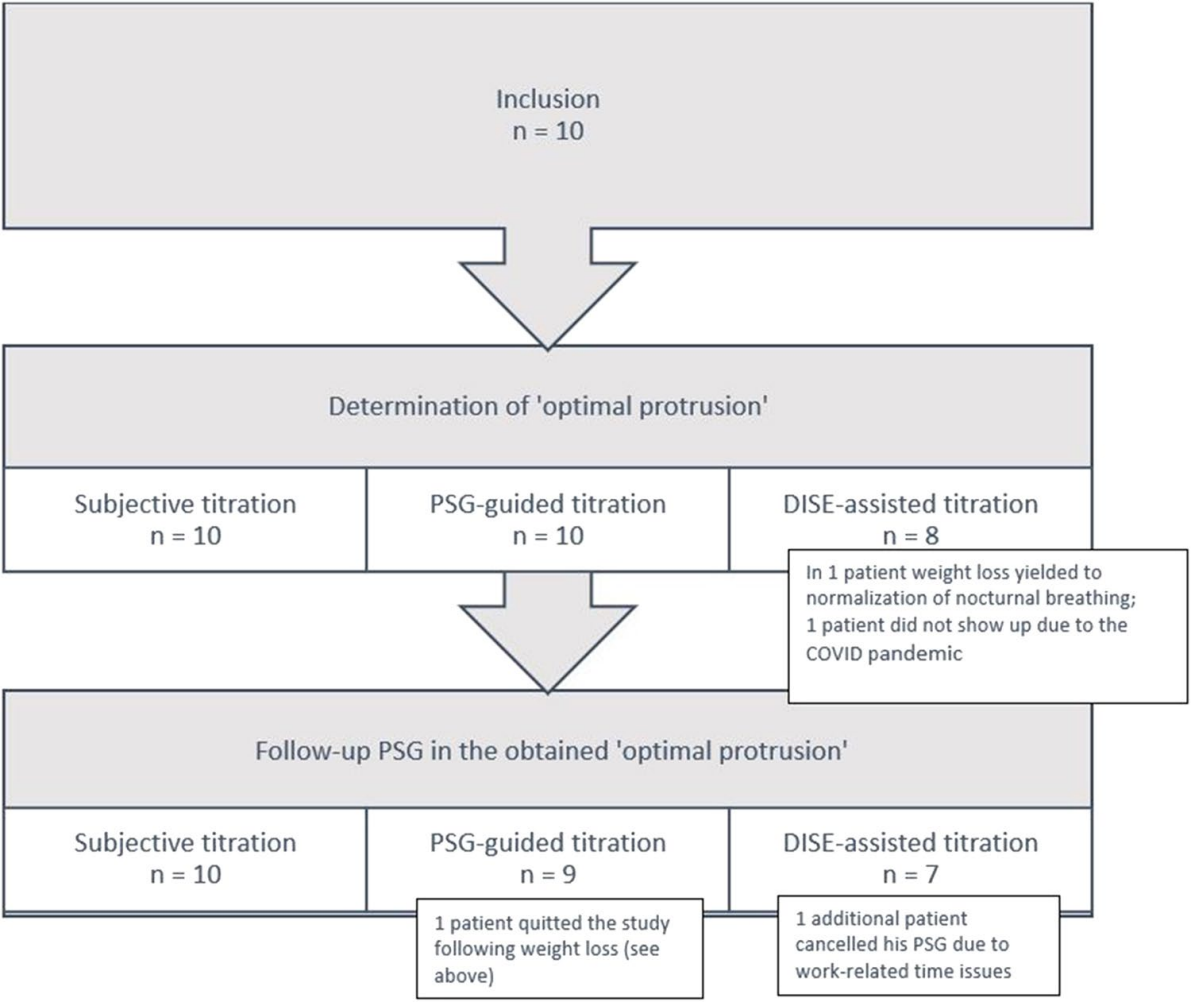
Flow chart of patients for the primary (optimal protrusion) and secondary outcome (efficacy of MAD therapy)

A flowchart of the study is shown in Fig. 2. The ‘optimal-protrusion’ could be determined in all patients for both subjective and titration_PSG_, and in 8 out of 10 patients for titration_DISE_. A follow-up PSG in the ‘optimal-protrusion’ was performed in all 10 patients after titration_Subj_, in 9 out of 10 patients after titration_PSG_ and in 7 out of 10 patients after titration_DISE_.

### Primary outcome: ‘optimal protrusion’

**Fig. 3:**
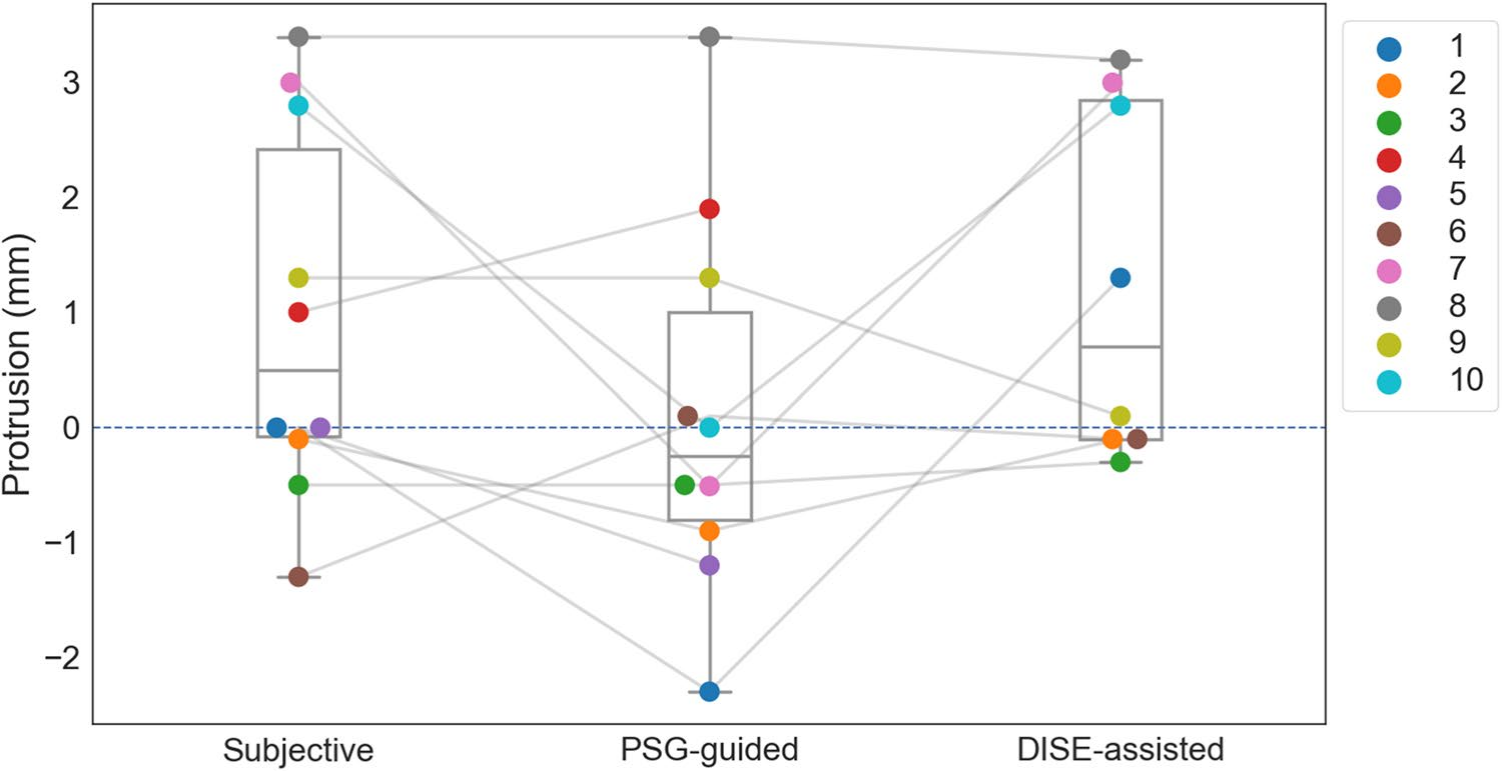
The ‘optimal protrusion’ relative to maximum comfortable protrusion (MCP) in each individual patient for the three different titration methods. Positive values represent a more forward mandibular position while negative values represent a more retruded position as compared to MCP

Overall, in 8 patients, the ‘optimal protrusion’ could be determined following all three titration methods. No significant differences in protrusion were demonstrated among the three different titration methods, with a median protrusion relative to MCP of 0.65 mm [− 0.2; 2.85], − 0.25 mm [− 0.6; 0.4] and 0.70 mm [− 0.1; 2.85] for titration_Subj_, titration_PSG_ and titration_DISE_, respectively (Fig. 3). In Table 2, details on the individual results are shown.

**Table 2:** Optimal protrusion obtained after the different titration methods, relative to maximum comfortable protrusion (MCP). *Q1* quantile 1, *Q3* quantile 3. Percentage of maximal protrusion is defined as the amount of protrusion relative to maximal possible protrusion of subjects

	Subjective	PSG-guided	DISE-assisted
Median titration (mm) [Q1; Q3]	0.65 [− 0.2; 2.85]	− 0.25 [− 0.6; 0.4]	0.70 [− 0.1; 2.85]
Optimal protrusion = MCP (%patients)	30	20	50
Optimal protrusion < MCP (%patients)	20	30	0
Optimal protrusion > MCP (%patients)	50	50	50
Percentage of maximal protrusion, % [Q1; Q3]	78.46 [71.5; 91.7]	73.6 [70.8; 77.5]	77.9 [74.3; 91.0]

Of eight patients, four patients were labelled ‘predicted success’ during the titration_PSG_, with 3 out of 4 (75%) being correctly classified as ‘responder’ (Table 3). On the other hand, three patients were labeled ‘predicted failure’ during the titration_PSG_ with all of them turning out to be ‘responder’ at the follow-up PSG with MAD. This equates to a sensitivity of 50% and a PPV of 75%. Both specificity and NPV are equal to 0%.

**Table 3:** Diagnostic accuracy of MAD treatment outcome prediction using RCMP during polysomnography and drug-induced sleep endoscopy

Actual treatment outcome	Predicted outcome during titration_PSG_		
	*Success*	*Failure*	*Inconclusive*
Responder	3	3	0
Non-responder	1	0	1
	4	3	1
	Predicted outcome during titration_DISE_		
Actual treatment outcome	*Success*	*Failure*	*Inconclusive*
Responder	5	1	0
Non-responder	0	1	0
	5	2	0

For titration_DISE_, five out of seven patients were scored with ‘predicted success’ and two with ‘predicted failure’. Overall, 6 out of 7 patients (85.7%) were correctly classified as ‘responder’ or ‘non-responder’. This equates to a sensitivity of 83.3%, a specificity of 100%, a PPV of 100% and a NPV of 50%.

### Secondary outcomes: efficacy of MAD therapy in terms of decrease in AHI, VAS for snoring, ESS and CIS20R

Seven patients went through all three study arms including the follow-up PSG in each optimal protrusion determined in each respective titration method.

**Fig. 4:**
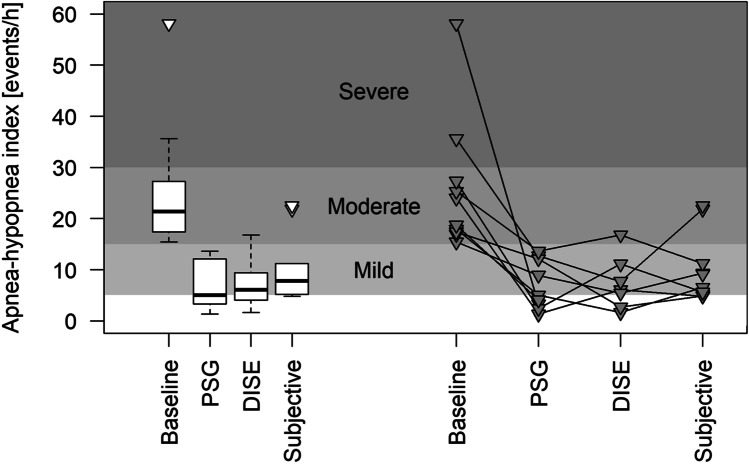
Evolution of apnea–hypopnea index (AHI) in comparison to baseline AHI in three different titration methods for 10 subjects. A few patients did not undergo a follow-up PSG after one or more titration method

Overall, the AHI decreased significantly from 24.0/h [17.4; 27.3] at baseline to 6.5/h [4.9; 11.2] after titration_Subj_, to 8.9/h [2.4; 12.7] after titration_PSG_ and to 6.1/h [2.7; 11.1] after titration_DISE_ (Fig. 4). There was no significant difference in the decrease in AHI according to the different titration procedures. When the decrease in AHI is presented in terms of the success definitions, for all three titration methods, 5 out of 7 subjects (71%) could be classified as a ‘responder’ with a reduction in AHI of 50% or more as compared to baseline, and/or a follow-up AHI of 10 events/hour or less.

In general, a relief in subjective complaints was observed: the VAS for snoring showed a reduction in severity of snoring, with on average silent snoring under MAD therapy for all three titration methods: VAS of 1 [1; 6], 4 [1; 4] and 4 [2; 9] after titration_Subj_, titration_DISE_, and titration_PSG_ respectively. Furthermore, the ESS decreased from 13 [10; 16] at baseline to 9 [3; 12] after titration_Subj_, 7 [6; 7] after titration_DISE_ and 10 [1; 10] after titration_PSG_, while the CIS20R decreased from 87 [41; 101] at baseline to 61 [43; 78] after titration_Subj_ and to 41 [41; 41] and 53 [42; 82] after DISE-assisted and titration_PSG_, respectively.

### Tertiary outcome: adherence to MAD therapy

Objective adherence data were available in nine patients during titration_Subj_, while objective adherence was measured in between the titration_PSG_ or the titration_DISE_ and the follow-up PSG in seven and five patients respectively. Data loss occurred due to lack of memory capacity on the chip or absence of the patient.

On average, the MAD was used for 4.1 ± 3.7 h per night during titration_Subj_, 3.8 ± 3.7 h per night after titration_PSG_ and 6.1 ± 3.1 h per night after titration_DISE_. Five patients (56%) could be considered ‘compliant’ with an average use of more than 4 h per night during titration_Subj_, while 43% of patients were ‘compliant’ after titration_PSG_ and 80% of patients met the definition of ‘compliant’ after titration_DISE_. All compliant patients fulfilled the criterion of ‘regular user’ as well (Table 4).

**Table 4:** Objectively measured adherence of mandibular advancement device (MAD) therapy in the different titration methods. Compliant user is defined as patients with MAD use for at least 4 h per night. Compliant user is defined as patients who use the MAD at least 4 h per night on at least 70% of all nights

	Titration_Subj_ *N* = 9	Titration_PSG_ *N* = 7	Titration_DISE_ *N* = 5
Mean wearing time (hours per night)	4.1 ± 3.7	3.8 ± 3.7	6.1 ± 3.1
Compliant users (%)	56	43	80
Regular users (%)	56	43	80

## Discussion

This pilot, randomized, cross-over clinical study was the first to compare different titration methods objectively to find the optimal mandibular position for MAD therapy. Overall, comparable amounts of titration and corresponding efficacy in terms of AHI reduction and reduction in subjective symptoms were found among the three titration methods.

On the one hand, in titration_Subj_, the relief of subjective complaints may lead to premature interruption of the titration and a suboptimal treatment outcome [29, 30]. On the other hand, objective titration may induce discomfort at the start of MAD treatment, therefore possibly making habituation more difficult.

Personalizing the mandibular protrusion in the individual patient, with respect to the patient’s physical limits, is imperative for successful treatment with MAD therapy [5, 17, 31]. In this process, there is no linear relationship between the amount of protrusion and AHI reduction [4–6]. The results of the present study confirm that titration is just a tool to achieve an optimal-protrusion, with negligible differences among the different titrations. However, both objectively guided methods yield a predicted starting position for MAD therapy, whereas titration_Subj_ lacks the predictive capacity of objective methods. The question remains whether the objective titration methods are capable of avoiding additional titration compared to titration_Subj_. On the other hand, finding an optimal-protrusion based on objective abolishment of upper airway collapse during DISE or respiratory events during PSG can act as starting point of titration_Subj_, requiring fine-tuning of the mandibular protrusion based on subjective complaints.

Sometimes, there is a need for substantial additional titration compared to MCP for all three titration protocols as was observed in one subject (#8). In the titration_PSG_ arm, five patients (50%) were reversed to a more retruded position relative to MCP, while this was not the case for the titration_DISE_. A possible explanation for this finding is that DISE is performed in supine position, which often triggers upper airway collapse with corresponding respiratory events. As such, titration_DISE_ may result in a ‘sleep-position independent mandibular protrusion’ by counteracting more effectively the generally detrimental effects of supine position on OSA. Therefore, future studies should consider this positional effect when comparing titration methods in determining the optimal-protrusion.

In terms of MAD efficacy, all three titrations significantly reduced AHI, with no significant difference between the methods. This finding emphasizes that similar optimal protrusive positions lead to similar therapeutic efficacy. This is further substantiated by the significant decrease in subjective parameters of VAS, ESS and CIS20R compared to baseline, again without differences between the titrations.

Titration_PSG_ correctly classified 50% of patients as ‘responder’, which is lower than the reported ranges of the literature (80 to 94%) [8, 20]. Besides the limited number of patients for the current study, there is yet no other explanation for this finding. A higher predictive accuracy was found for titration_DISE_ with a sensitivity of 83.3%, a specificity of 100%, a PPV of 100% and a NPV of 50%.

Patients labelled as ‘predicted failure’ during titration_PSG_ (*n* = 3) or titration_DISE_ (*n* = 2) were all fitted with an MAD locked in 75% of the MP prior to the respective follow-up PSG. For titration_PSG_, all three patients showed a successful treatment response in 75% of MP at the follow-up PSG. For titration_DISE_, one patient showed a successful treatment response in the 75% of MP, with no improvement in the other patient. So, in about one-third of the study population, there is negligible improvement of OSA, which is in line with recent findings [32]. Supposedly, in this segment of patients, treatment response and sensitivity to predictive investigations are rather unpredictable. Future research should aim at a full exploration of such patients in order to understand the possible underlying mechanisms of non-response.

The objectively measured adherence proved that 56% of patients used the MAD more than 4 h per night on average during titration_Subj_, while 43% of patients were compliant after titration_PSG_ and 80% of patients after titration_DISE_. Due to the limited number of patients, objective adherence data were only available in four patients for all three titration methods studied, revealing no difference in adherence among the protocols. However, one could hypothesize that similar optimal protrusive positions in terms of efficacy will lead to similar adherence regardless of the protocol.

The strength of our study is the prospective crossover design. This allows for within-subject analyses, thereby reducing the errors caused by the individual differences. The weakness of our study is the intensive study design for both patients and investigators, thereby limiting the number of included patients. Further studies, preferably in a multi-center setting, with a larger sample size with a longer follow-up period are the solution to increase inclusion and to validate the results of the current investigation. Furthermore, new advances in technology in terms of a feedback-controlled mandibular positioner that identifies respiratory events in real time at home while protruding the mandible [33] could even take the overnight titration out of the hospital environment into the patients’ everyday environment.

Overall, this pilot prospective, randomized, crossover clinical trial showed no differences in optimal mandibular protrusion and corresponding efficacy of MAD after titration_Subj_, titration_DISE_ or titration_PSG_ in the individual patient. The results of this study are a step forward in the personalized management of targeting the optimal mandibular protrusion in the individual patient.

## Data Availability

The datasets generated during and/or analyzed during the current study are available from the corresponding author on reasonable request.

## Abbreviations

AHI: Apnea-hypopnea index
BMI: Body mass index
CPAP: Continuous positive airway pressure
DISE: Drug-induced sleep endoscopy
EDS: Excessive daytime sleepiness
ESS: Epworth Sleepiness Scale
MAD: Mandibular advancement device
MCP: Maximum comfortable protrusion
MP: Maximum protrusion
OSA: Obstructive sleep apnea
PSG: Polysomnography
RCMP: Remotely controlled mandibular positioner
VAS: Visual analog scale
